# Cerebral metabolite alterations in patients with posttransplant encephalopathy after liver transplantation

**DOI:** 10.1371/journal.pone.0221626

**Published:** 2019-08-23

**Authors:** Henning Pflugrad, Anita Blanka Tryc, Annemarie Goldbecker, Hannelore Barg-Hock, Christian Strassburg, Jürgen Klempnauer, Heinrich Lanfermann, Karin Weissenborn, Peter Raab

**Affiliations:** 1 Department of Neurology, Hannover Medical School, Hannover, Germany; 2 Integrated Research and Treatment Centre Transplantation, Hannover, Germany; 3 Clinic for Visceral and Transplant Surgery, Hannover Medical School, Hannover, Germany; 4 Department of Gastroenterology, Hepatology and Endocrinology, Hannover Medical School, Hannover, Germany; 5 Institute of Diagnostic and Interventional Neuroradiology, Hannover Medical School, Hannover, Germany; University at Buffalo, UNITED STATES

## Abstract

**Background:**

In the first weeks after liver transplantation about 30% of the patients develop a posttransplant encephalopathy. A posttransplant encephalopathy comprises metabolic-toxic caused symptoms such as disorientation, confusion, hallucinations, cognitive dysfunction and seizures. We hypothesize that alterations of cerebral metabolites before liver transplantation predispose posttransplant encephalopathy development after liver transplantation.

**Methods:**

31 patients with chronic liver disease underwent magnetic resonance spectroscopy (MRS) before liver transplantation to assess glutamine/glutamate (Glx), myo-Inositol (mI), choline (Cho), creatine/phosphocreatine- and N-acetyl-aspartate/N-acetyl-aspartate-glutamate concentrations in the thalamus, lentiform nucleus and white matter. Of these, 14 patients underwent MRS additionally after liver transplantation. Furthermore, 15 patients received MRS only after liver transplantation. Patients’ data were compared to 20 healthy age adjusted controls.

**Results:**

Patients showed significantly increased Glx and decreased mI and Cho concentrations compared to controls before liver transplantation (p≤0.01). The MRS values before liver transplantation of patients with posttransplant encephalopathy showed no significant difference compared to patients without posttransplant encephalopathy. Patients after liver transplantation showed increased Glx concentrations (p≤0.01) compared to controls, however, patients with and without posttransplant encephalopathy did not differ. Patients with posttransplant encephalopathy who underwent MRS before and after liver transplantation showed a significant mI increase in all three brain regions (p<0.04) and Glx decrease in the lentiform nucleus after liver transplantation (p = 0.04) while patients without posttransplant encephalopathy only showed a mI increase in the thalamus (p = 0.04).

**Conclusion:**

Patients with and without posttransplant encephalopathy showed no significant difference in cerebral metabolites before liver transplantation. However, the paired sub-analysis indicates that the extent of cerebral metabolite alterations in patients with liver cirrhosis might be critical for the development of posttransplant encephalopathy after liver transplantation.

## Introduction

Neurological complications within the first weeks after orthotopic liver transplantation (OLT) are frequent [1]. Up to 30% of the patients develop central nervous system (CNS) complications such as seizures, confusion, hallucinations, disorientation, clouding of consciousness, central pontine myelinolysis or CNS infections [2]. Structural brain damage is rare in these patients, thus, in most cases a metabolic-toxic cause, classified as posttransplant encephalopathy (PTE), is assumed [2–4]. PTE is a severe neurological complication which leads to a prolonged stay on the intensive care unit and increases length of hospitalization [1]. Fortunately, PTE is not associated with increased mortality or long-term neurological impairment [3]. The pathomechanism of PTE is unknown so far. Discussed are extensive cerebral metabolite changes, electrolyte shift and dysbalance of water homeostasis during and after surgery as well as neurotoxicity of calcineurin inhibitors (CNI) [2, 5–8].

Changes in cerebral metabolites have been detected in deep grey matter nuclei and white matter in patients with liver cirrhosis with and without hepatic encephalopathy (HE) using proton magnetic resonance spectroscopy (^1^H-MRS) [9–11]. The patients show a decreased myo-Inositol/creatine and choline/creatine as well as an increased glutamine and glutamate (Glx)/creatine ratio compared to controls [10]. These alterations were shown to normalize after OLT [9, 12, 13].

PTE induced neurological impairment seems to be only transient, thus, cerebral metabolite changes associated with liver cirrhosis and/or HE might be involved in the development of PTE after OLT. However, it is unknown so far whether cerebral MRS alterations before OLT might be related to the development of PTE. Furthermore, the course of cerebral MRS alterations after OLT was not addressed so far in patients with and without PTE.

We hypothesized that patients with PTE in the first weeks after OLT show significantly altered MRS values in deep grey matter nuclei and white matter before OLT compared to patients without PTE and healthy controls. Furthermore, we hypothesized that patients who developed PTE show more pronounced changes of the brain metabolites between baseline and follow up examination after OLT than patients without PTE.

## Patients and methods

### Patients and controls

The patients included into this analysis took part in a long-term follow up study of patients after liver transplantation performed at Hannover Medical School, Hannover, Germany. For a detailed description of the patient cohort see [14].

**Fig 1:**
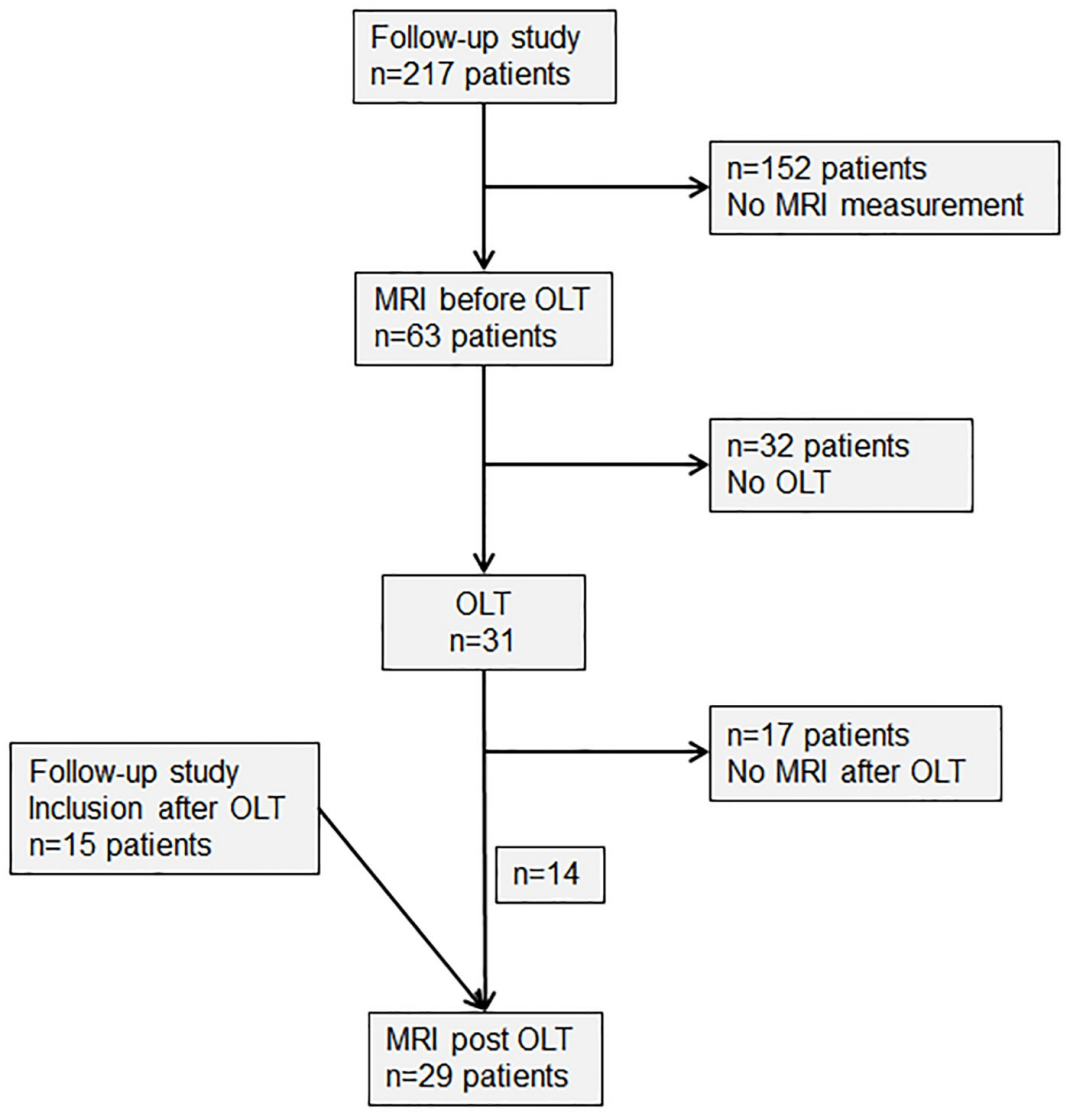
Flow chart. This flow chart displays the application of the exclusion criteria. MRI, magnetic resonance imaging; OLT, liver transplantation; n, number.

Between September 2008 and October 2014 217 patients listed for liver transplantation at Hannover Medical School agreed to participate in a follow up study including the time on the waiting list as well as follow-up after OLT. 195 (89.9%) of these patients had liver cirrhosis. 63 of the cirrhotic patients underwent magnetic resonance imaging (MRI) and proton magnetic resonance spectroscopy (^1^H-MRS) of the brain. 31 of these 63 patients (age 47.8±12.3 years, 20 (65%) male) received OLT 10.9±16.5 months after MRI/MRS and were finally considered for the present analysis. Fourteen (45%) were available for follow-up MRI/MRS 12.1±3.6 months after OLT. Reasons for missing follow-up measurement after OLT in the other patients were death (n = 7) or decline of participation (n = 10). Another 15 patients who underwent OLT for liver cirrhosis could be studied by MRI/MRS for the first time 17.6±7.5 months after OLT. Thus, in total MRS data were available from 31 patients before OLT, 14 patients before and after OLT and 29 patients after OLT (Fig 1).

The patients were subdivided according to the occurrence of a PTE in the first weeks after OLT. Of the 31 patients who underwent MRI before OLT three died after OLT while still under general anaesthesia and thus could not be included in the analysis. Nine of the remaining 28 patients (32%) developed PTE. The neurological symptoms were disorientation (n = 4), confusion (n = 2), confusion and somnolence (n = 1), hallucinations (n = 1) and somnolence (n = 1).

Of the 29 patients who underwent MRI/MRS after OLT 10 (34%) had developed PTE. The patients presented with disorientation (n = 3), confusion (n = 3), confusion and somnolence (n = 2) and hallucinations (n = 2).

Within the subgroup of patients (n = 14) who underwent MRI before and after OLT 7 (50%) developed a PTE.

Patients’ MRS findings were compared to those of 20 healthy age adjusted controls (age 47.8±10.8 years, 6 (30%) male). Exclusion criteria for this study were neurological or psychiatric diseases not related to hepatic encephalopathy, additional transplantation of another organ, history of liver transplantation or liver re-transplantation >3 months after the first OLT, contraindications for MRI, acute liver failure, chronic liver disease without liver cirrhosis and regular intake of medication affecting brain function.

All subjects gave written informed consent. The study was approved by the local ethics committee at Hannover Medical School and performed according to the World Medical Association Declaration of Helsinki (revised in 2008). No organs from executed prisoners were used. None of the transplant donors were from a vulnerable population and all donors or next of kin provided written informed consent that was freely given.

### Methods

Before OLT patients underwent neurological examinations every 6 months. After transplantation they were examined on days 1, 7 and 90 and additionally if needed due to neurological complications by a neurologist of the group. Patients with neurological complications after OLT were diagnosed with PTE if the diagnostic work-up–including laboratory testing, brain imaging, electroencephalography and eventually also lumbar puncture—excluded other reasons for their symptoms and a metabolic-toxic cause was assumed. The patients were subdivided into patients with PTE (group 1) and patients without PTE (group 2).

Age, sex, liver disease underlying cirrhosis, arterial hypertension (AH), diabetes mellitus (DM), hypercholesterolemia (HC), glomerular filtration rate (GFR) in ml/min at the time of MRI/MRS examination, laboratory Model of End Stage Liver Disease (labMELD) score directly before OLT, immunosuppression and other medication, history of hepatic encephalopathy (HE), HE grade according to the West Haven Criteria (WHC) at the time points of MRS measurement and OLT, respectively, number of additional surgical interventions after OLT, reason for OLT (chronic or acute-on-chronic liver failure[15] (ACLF) and survival were registered. 29 (63%) of the 46 patients included into this study received OLT according to matchMELD which considers severe comorbidities and subsequently results in higher MELD scores.

#### Magnetic resonance spectroscopy

**Fig 2:**
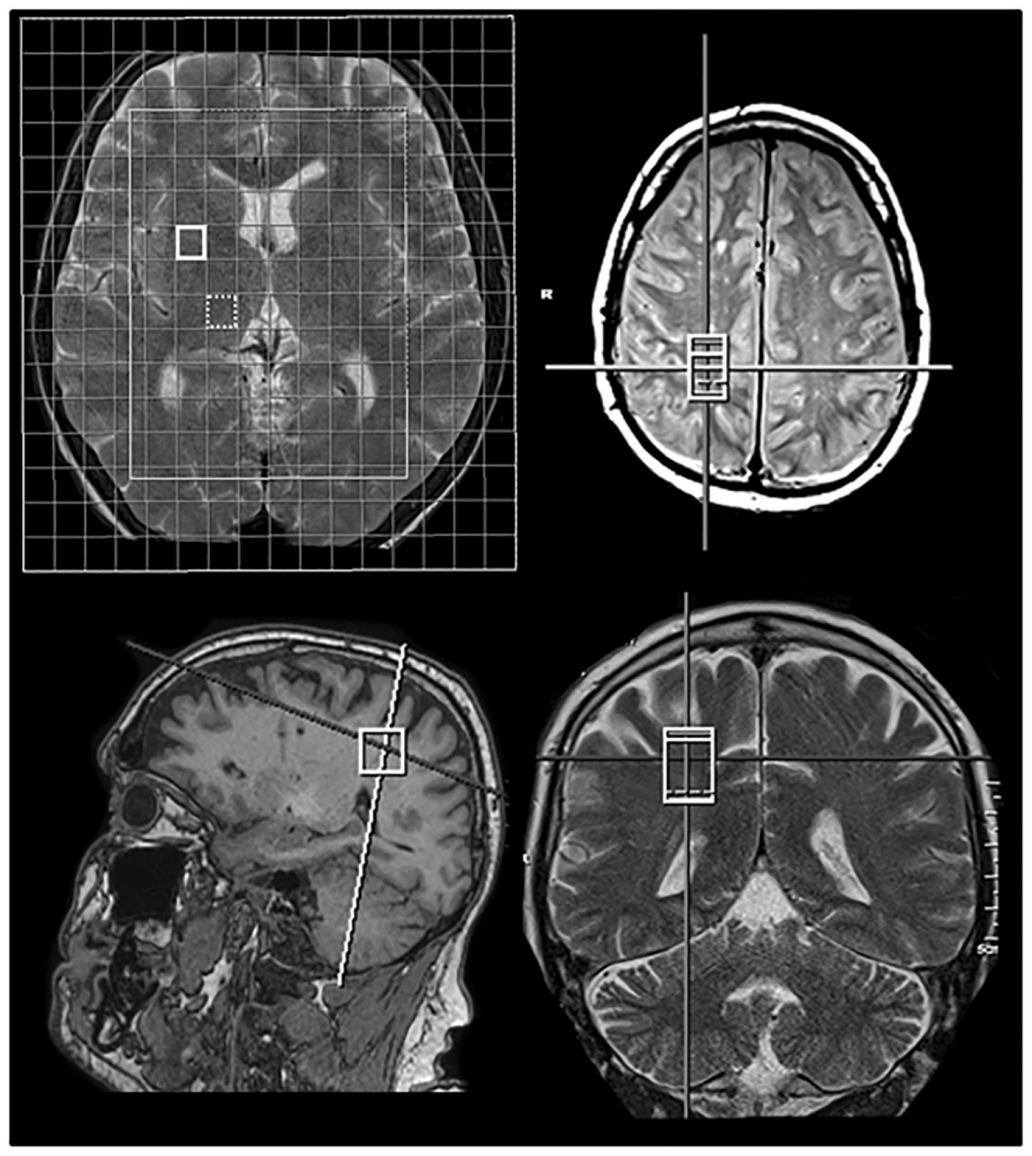
Voxel positions. Illustration of the positions of the 2D-CSI-Grid, indicating the voxel position within the putamen (solid white box) and the thalamus (dashed white box). Triplanar illustration of the position of the single voxel spectroscopy within the parietal white matter, avoiding cerebrospinal fluid.

**Fig 3:**
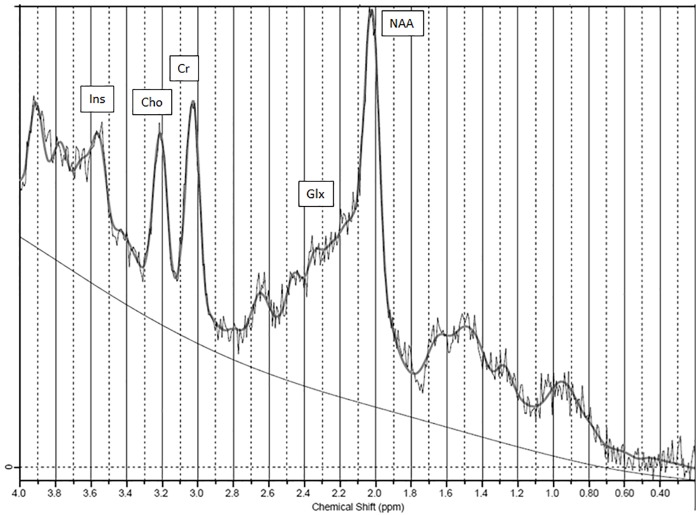
Example of metabolite spectrums of a patient. Example of the LCModel-analysis of a thalamic 2D-CSI voxel acquired with TE 30ms (TE = time of echo) in a patient. The grey line following the peaks represents the fit by the program, the thin line below the spectrum indicates the baseline calculated by the LCModel program. Ins = inositol peak, Cho = choline peak, Cr = creatine/phosphocreatine peak, Glx = glutamate/glutamine peaks, NAA = N-acetyl-aspartate peak.

^1^H-MRS was performed at a 1.5 Tesla scanner (AVANTO, Siemens Erlangen, Germany) using the standard 4 channel receiving head matrix coil. Spectroscopy sequence positioning was performed by a senior neuroradiologist with special focus on minimizing partial volume effects like avoiding ventricles and grey matter. The MRS-protocol consisted of multiplanar T2-sequences for planning purposes (axial T2-sequence: repetition time (TR) 3100ms, echo time (TE) 134ms, slice thickness 5mm, pulse angle 150; sagittal T2-sequence: TR 5150ms, TE 107ms, slice thickness 3mm; coronal T2-sequence: TR 4500ms, TE 93ms, slice thickness 5mm) and one single voxel spectroscopy-measurement using the Point-resolved spectroscopy (PRESS) volume selection (parietal white matter (pWM); 8ml, TR 3000ms, TE 30ms, 96 acquisitions, water suppression was achieved using chemical shift-selective saturation pulses (CHESS); additional acquisition of the unsuppressed water signal using the same sequence with only 16 acquisitions in the same location). A second spectroscopy sequence using a 2D-PRESS-chemical shift imaging method was performed, additionally (field of view 160x160, matrix 16x16, TR 1500ms, TE 30ms, number of averages 4, slice thickness 1,5cm, water suppression using CHESS pulses, slice angulation from the middle of the head of caudate to pulvinar thalami also containing the lentiform nucleus, scanning time 7:22min; there was no acquisition with unsuppressed water signal for this 2D-sequence). Shimming for the spectroscopy sequences was done automatically by the MR scanner, leading to Full Width at Half Maximum (FWHM) line widths of 0.06–0.18 ppm. Raw data of the spectra were analysed using LCModel (version 6.2 [16]) in order to get arbitrary units for the metabolite concentrations, the single-voxel spectroscopy data were referenced to the unsuppressed water signal by the LCModel software. There was no correction for possible different receiver gains between patients and partial volume correction to adjust for cerebrospinal fluid and grey matter was not performed. From the 2D-chemical shift imaging sequence voxels containing the right thalamus (Th) and the right anterior lentiform (LN) nucleus were selected for analysis using LCModel without referencing to an unsuppressed water signal. The positions of the voxels are displayed in Fig 2. To assure a reliable quality of the MRS data only measurements with a standard error estimate (Cramer-Rao lower bound, as indicated by the LCModel software) below 20% were considered for the analysis. Thus, the MRS pWM values in 2 patients before OLT, 1 patient after OLT and 3 controls had to be excluded from the analysis. The concentrations of glutamine/glutamate (Glx)-, myo-Inositol (mI)-, choline (Cho)-, creatine/phosphocreatine (Cr)- and N-acetyl-aspartate/N-acetyl-aspartate-glutamate (NAA) were measured. NAA is a surrogate marker for neuronal health as it indicates neuronal cell density and function. Cho is associated with cellular membrane turnover and Cr is part of the energy metabolism by the creatine kinase reaction. mI is part of the intracellular inositol triphosphate system and serves as cellular osmolyte. Glx consists of glutamate (Glu) and glutamine (Gln) which cannot be separated at a 1.5 Tesla MRI. Glu is an excitatory neurotransmitter and Gln is involved in protein synthesis [17]. Fig 3 illustrates an example of a metabolite spectrum of a patient.

### Statistical methods

Normality of distribution was tested by Shapiro-Wilk test. Group differences for abnormally distributed values were tested by Kruskal-Wallis test and Mann-Whitney U test. For normally distributed values Student’s t-test or analysis of variance (ANOVA) with Bonferroni or Dunnett-T test according to variance homogeneity were applied. The assessment of the dependent MRS measurements performed before and after OLT was done by Wilcoxon signed-rank test. For the analysis of the paired MRS measurements the metabolite/creatine ratio was calculated for each metabolite additionally. Categorical variables were tested by chi-squared (χ2) test. Correlation analysis was performed with the Pearson test (normally distributed values) and the Spearman’s rank test (abnormally distributed values). Normal distributed values are listed as mean ± standard deviation (SD), abnormal distributed values are shown as median with interquartile range (IQR). A p-value <0.05 was considered significant for all tests applied. Statistical analysis was performed using SPSS, Version 24 (IBM, Armonk, New York, USA).

## Results

### MRS results achieved before OLT analysed in regard to the development of PTE thereafter

31 patients (age 47.8±12.3 years, 64.5% men) underwent MRI 10.9±16.5 months before OLT. Patients and controls did not differ concerning age, however, men predominated in the patient group (p = 0.02). The details of the patient group characteristics are displayed in Table 1. Patients had significantly lower levels of mI (p<0.001) in the thalamus, lentiform nucleus and parietal white matter as well as significantly increased levels of Glx (p<0.001) compared to healthy controls. Furthermore, Cho levels were significantly lower in patients in the thalamus (p = 0.01) and white matter (p = 0.001) compared to controls while the Cr level in the lentiform nucleus was significantly increased (p<0.001). Patients with diabetes (n = 4) showed a significantly lower mI level in the white matter than patients without DM (p = 0.01), however, due to the low patient number with diabetes this result is not representative. Other diseases (aHT, HC and CKD ≥ grade 3) showed no effect on MRS results, nor did a history of HE or the WHC grade of HE at the time of the examination. Correlation analysis showed a significant negative correlation between age and thalamic mI (-0.37, p = 0.04; n = 31) as well as white matter mI (-0.46, p = 0.01; n = 30) in the patients. In contrast, healthy controls showed a significant positive correlation between age and white matter mI (0.75, p = 0.001; n = 17).

**Table 1:** Patient characteristics and MRS values before OLT compared to controls.

	Patients n = 31	Controls n = 20	p-value
**Age mean±SD**	47.8±12.3	47.8±10.8	0.98
**Gender male/female%**	20(65%)/11	6(30%)/14	**0.02**
**Underlying liver disease**	HCV n = 7 (23%) HBV n = 4 (13%) AIH n = 8 (25%) AC n = 5 (16%) Other n = 7 (23%)	na	-
**AH y/n %**	4(13%)/27	na	-
**DM y/n %**	4(13%)/27	na	-
**HC y/n %**	1(3%)/30	na	-
**GFR ml/min mean±SD**	100±34.2	-	-
**CKD ≥grade 3 y/n %**	5(16%)/26	na	-
**labMELD at OLT mean±SD**	15.8±6.2	na	-
**matchMELD mean±SD**	29.8±5.0 (n = 23)	na	-
**History of HE y/n %**	11(36%)/20	na	-
**WHC Grade %** **at MRS measurement**	0 n = 27 (87%) 1 n = 4 (13%)	na	-
**Th Cho mean±SD**	0.91±0.13	1.02±0.14	**0.01**
**Th Cr mean±SD**	3.61±0.50	3.41±0.39	0.13
**Th NAA mean±SD**	4.92±0.86	5.04±0.86	0.63
**Th mI mean±SD**	1.61±0.73	2.72±0.32	**<0.001**
**Th Glx median (IQR)**	7.70 (2.44)	5.72 (1.86)	**<0.001**
**Ln Cho median (IQR)**	0.99 (0.36)	1.00 (0.12)	0.91
**Ln Cr mean±SD**	4.32±0.49	3.83±0.36	**<0.001**
**Ln NAA mean±SD**	5.12±0.71	4.96±0.47	0.39
**Ln mI median (IQR)**	1.85 (1.31)	2.72 (0.39)	**<0.001**
**Ln Glx mean±SD**	9.37±1.74	6.57±0.86	**<0.001**
**pWM Cho mean±SD**	0.94±0.14 (n = 29)	1.08±0.15 (n = 17)	**0.001**
**pWM Cr median (IQR)**	3.30 (0.50) (n = 29)	3.23 (0.26) (n = 17)	0.30
**pWM NAA median (IQR)**	5.72 (0.72) (n = 29)	5.60 (0.69) (n = 17)	0.26
**pWM mI mean±SD**	1.43±0.85 (n = 29)	3.08±0.63 (n = 17)	**<0.001**
**pWM Glx median (IQR)**	5.90 (1.91) (n = 29)	4.91 (0.97) (n = 17)	**<0.001**

MRS, magnetic resonance spectroscopy; OLT, liver transplantation; SD, standard deviation; n, number; HCV, Hepatitis C Virus; HBV, Hepatitis B Virus; AIH, Autoimmune Hepatitis; AC, alcoholic cirrhosis; AH, arterial hypertension, DM, diabetes mellitus, HC, hypercholesterolaemia; CKD, chronic kidney disease grade 3 according to GFR, glomerular filtration rate at the time of MRI/MRS examination; labMELD, laboratory Model of End Stage Liver Disease score; HE, hepatic encephalopathy; WHC, West Haven Criteria; Th, Thalamus; Ln, Lentiform nucleus; pWM, parietal white matter; Cho, choline; Cr, creatine; Naa, N-acetyl-aspartate; mI, myo-Inositol; Glx, glutamine/glutamate; y, yes; n, no; IQR, interquartile range; na, not applicable; p value ≤0.05 is considered significant

The patients were subdivided according to the development of PTE after OLT. Since 3 patients died after OLT while still hold under general anaesthesia only 28 patients could be considered for this analysis. 9 of these 28 patients (50.7±11.3 years, 89% male) developed a PTE, while 19 (47.4±12.3 years, 47% male) did not. Thus, the PTE subgroup differed from the Non-PTE group in regard to sex distribution (p = 0.01). In addition the PTE patients underwent significantly more additional surgical interventions after OLT than those without PTE (p = 0.04). Otherwise the two groups showed no significant differences in baseline characteristics (Table 2). Both patient groups showed significantly lower levels of mI (p≤0.001) and increased levels of Glx (p<0.01) in the thalamus, lentiform nucleus and white matter compared to controls. Cr concentrations were significantly higher in patients without PTE than in controls in the thalamus (p = 0.04) and lentiform nucleus (p<0.001) while Cho was significantly lower (p = 0.003) in the white matter compared to controls (Table 2). Myo-inositol was lowest and Glx highest in all brain regions in the PTE-group compared to patients without PTE and controls. The difference between the two patient groups, however, missed the level of significance probably due to the small patient numbers.

**Table 2:** Characteristics and MRS values before OLT of patients with and without PTE.

	Patients with PTE n = 9 (Group 1)	Patients no PTE n = 19 (Group 2)	Controls n = 20 (Group 3)	p-value
**Age mean±SD**	50.7±11.3	47.4±12.3	47.8±10.8	0.76
**Gender male/female%**	8(89%)/1	9(47%)/10	6(30%)/14	*0.01
**Underlying liver disease**	HCV n = 2 (22%) HBV n = 0 AI n = 2 (22%) AC n = 3 (34%) Other n = 2 (22%)	HCV n = 4 (21%) HBV n = 4 (21%) AI n = 5 (26%) AC n = 2 (11%) Other n = 4 (21%)	na	0.45
**Reason for OLT %**	chronic n = 7 (78%) ACLF n = 2 (22%)	chronic n = 18 (95%) ACLF n = 1 (5%)	na	0.23
**Additional surgery y/n %**	8(89%)/1	8(42%)/11	na	**0.04**
**AH y/n %**	2(22%)/7	2(11%)/17	na	0.57
**DM y/n %**	2(22%)/7	2(11%)/17	na	0.57
**HC y/n %**	1(11%)/8	0(0%)/19	na	0.32
**GFR ml/min mean±SD**	85.4±11.0	100.7±31.5	-	0.25
**CKD ≥grade 3 y/n %**	3(33%)/6	2(11%)/17	na	0.29
**labMELD at OLT mean±SD**	19.9±4.5	13.9±4.5	na	0.96
**HE y/n %**	4(44%)/5	7(37%)/12	na	1.00
**WHC Grade %** **at MRS measurement**	0 n = 7 (78%) 1 n = 2 (22%)	0 n = 17 (90%) 1 n = 2 (10%)	na	0.57
**WHC Grade %** **at OLT**	0 n = 7 (78%) 1 n = 2 (22%)	0 n = 16 (85%) 1 n = 1 (5%) 2 n = 1 (5%) 3 n = 1 (5%)	na	0.45
**IS Tac/CyA %** **after OLT**	5(56%)/4	13(68%)/6	na	0.68
**Th Cho mean±SD**	0.90±0.12	0.94±0.13	1.02±0.14	0.05
**Th Cr mean±SD**	3.47±0.48	3.77±0.47	3.41±0.39	*0.03 **Group 2 vs Group 3** **0.04**
**Th NAA mean±SD**	4.79±1.08	5.10±0.71	5.04±0.86	0.66
**Th mI mean±SD**	1.44±0.54	1.65±0.85	2.72±0.32	*<0.001 **Group 1 vs Group 3 <0.001** **Group 2 vs Group 3 <0.001** **Group** 1 vs **Group** 2 p = 1.00
**Th Glx median (IQR)**	7.95 (3.54)	7.70 (2.15)	5.72 (1.86)	*<0.001 **Group 1 vs Group 3 <0.01** **Group 2 vs Group 3 = 0.001** **Group** 1 vs **Group** 2 p = 1.00
**Ln Cho median (IQR)**	0.96 (0.28)	1.00 (0.30)	1.00 (0.12)	0.32
**Ln Cr mean±SD**	4.17±0.34	4.45±0.53	3.83±0.36	*<0.001 **Group 2 vs Group 3 <0.001**
**Ln NAA mean±SD**	5.02±0.60	5.14±0.74	4.96±0.47	0.68
**Ln mI median (IQR)**	1.32 (1.26)	1.81 (1.59)	2.72 (0.39)	*<0.001 **Group 1 vs Group 3 <0.01** **Group 2 vs Group 3 <0.01** **Group** 1 vs **Group** 2 p = 1.00
**Ln Glx mean±SD**	10.41±1.90	9.07±1.57	6.57±0.86	*<0.001 **Group 1 vs Group 3 <0.001** **Group 2 vs Group 3 <0.001** **Group** 1 vs **Group** 2 p = 0.80
**pWM Cho mean±SD**	0.98±0.13	0.92±0.13 (n = 17)	1.08±0.15 (n = 17)	*<0.01 **Group 2 vs Group 3 <0.01**
**pWM Cr median (IQR)**	3.34 (0.30)	3.25 (0.58) (n = 17)	3.23 (0.26) (n = 17)	0.71
**pWM NAA median (IQR)**	5.98 (0.73)	5.59 (0.71) (n = 17)	5.60 (0.69) (n = 17)	0.37
**pWM mI mean±SD**	1.13±0.95	1.54±0.85 (n = 17)	3.08±0.63 (n = 17)	*<0.001 **Group 1 vs Group 3 <0.001** **Group 2 vs Group 3 <0.001** **Group** 1 vs **Group** 2 p = 0.65
**pWM Glx median (IQR)**	6.65 (5.69)	5.83 (1.45) (n = 17)	4.91 (0.97) (n = 17)	*<0.001 **Group 1 vs Group 3 <0.001** **Group 2 vs Group 3** **<0.01** **Group** 1 vs **Group** 2 p = 0.58

MRS, magnetic resonance spectroscopy; OLT, liver transplantation; PTE, post-transplant encephalopathy; SD, standard deviation; n, number; HCV, Hepatitis C Virus; HBV, Hepatitis B Virus; AIH, Autoimmune Hepatitis; ACLF, acute-on-chronic liver failure; AC, alcoholic cirrhosis; AH, arterial hypertension, DM, diabetes mellitus, HC, hypercholesterolaemia; CKD, chronic kidney disease grade 3 according to GFR, glomerular filtration rate at the time of MRI/MRS examination; labMELD, laboratory Model of End Stage Liver Disease score; HE, hepatic encephalopathy; WHC, West Haven Criteria; IS, immunosuppression; Tac, Tacrolimus; CyA, Cyclosporine A; Th, Thalamus; Ln, Lentiform nucleus; pWM, parietal white matter; Cho, choline; Cr, creatine; Naa, N-acetyl-aspartate; mI, myo-inositol; Glx, glutamine/glutamate; y, yes; n, no; IQR, interquartile range; na, not applicable; p value ≤0.05 is considered significant;

*, overall between groups

### MRS results achieved months after OLT analysed in regard to prior development of PTE

29 patients (age 52.2 ± 9.4 years, 89.7% male) underwent MRI 15.0±6.5 months after OLT. Patients and controls did not differ concerning age, however, again not surprisingly the patient group consisted of more men (p<0.001). The details of the patient group characteristics are displayed in Table 3. Interestingly, Glx levels were still significantly increased in the thalamus (p = 0.01) and lentiform nucleus (p = 0.001) in patients compared to controls. However, the Glx levels were lower than those measured in the patients awaiting OLT. Furthermore, Cho levels were significantly higher in patients than in controls in the lentiform nucleus (p<0.01). Metabolite levels in the white matter did not differ between control and patients after OLT. Other diseases (DM, aHT, HC and CKD ≥ grade 3) showed no effect on MRS parameters, neither did a history of HE. No correlations were found between MRS parameters of the patients and age and GFR at the time of MRI/MRS examination.

**Table 3:** Patient characteristics and MRS values after OLT compared to controls.

	Patients n = 29	Controls n = 20	p-value
**Age mean±SD**	52.2±9.4	47.8±10.8	0.13
**Gender male/female%**	26(81%)/3	6(30%)/14	**<0.001**
**Underlying liver disease**	HCV n = 4 (14%) HBV n = 8 (28%) AI n = 4 (14%) AC n = 5 (16%) Other n = 8 (28%)	na	-
**AH y/n %**	11(38%)/18	na	-
**DM y/n %**	5(17%)/24	na	-
**HC y/n %**	2(7%)/27	na	-
**GFR ml/min mean±SD**	77.3±35.0	-	-
**CKD ≥grade 3 y/n %**	10(35%)/19	na	-
**labMELD at OLT mean±SD**	19.4±9.6	na	-
**matchMELD mean±SD**	29.5±4.7	na	-
**History of HE y/n %**	17(59%)/12	na	-
**IS Tac/CyA %** **after OLT**	16(55%)/13	na	-
**Th Cho mean±SD**	1.11±0.20	1.02±0.14	0.08
**Th Cr mean±SD**	3.34±0.38	3.41±0.39	0.57
**Th NAA mean±SD**	4.90±0.84	5.04±0.86	0.59
**Th mI mean±SD**	2.71±0.50	2.72±0.32	0.90
**Th Glx median (IQR)**	6.90 (1.55)	5.72 (1.86)	**0.01**
**Ln Cho median (IQR)**	1.26 (0.45)	1.00 (0.12)	**0.02**
**Ln Cr mean±SD**	3.99±0.82	3.83±0.36	0.43
**Ln NAA mean±SD**	5.02±0.81	4.96±0.47	0.79
**Ln mI median (IQR)**	2.82 (0.86)	2.72 (0.39)	0.78
**Ln Glx mean±SD**	7.92±1.81	6.57±0.86	**0.001**
**pWM Cho mean±SD**	1.05±0.25 (n = 28)	1.08±0.15 (n = 17)	0.70
**pWM Cr median (IQR)**	3.26 (0.47) (n = 28)	3.23 (0.26) (n = 17)	0.48
**pWM NAA median (IQR)**	5.37 (1.01) (n = 28)	5.60 (0.69) (n = 17)	0.31
**pWM mI mean±SD**	2.85±1.06 (n = 28)	3.08±0.63 (n = 17)	0.37
**pWM Glx median (IQR)**	5.06 (2.07) (n = 28)	4.91 (0.97) (n = 17)	0.96

MRS, magnetic resonance spectroscopy; OLT, liver transplantation; SD, standard deviation; n, number; HCV, Hepatitis C Virus; HBV, Hepatitis B Virus; AIH, Autoimmune Hepatitis; AC, alcoholic cirrhosis; AH, arterial hypertension, DM, diabetes mellitus, HC, hypercholesterolaemia; CKD, chronic kidney disease grade 3 according to GFR, glomerular filtration rate at the time of MRI/MRS examination; labMELD, laboratory Model of End Stage Liver Disease score; HE, hepatic encephalopathy; WHC, West Haven Criteria; IS, immunosuppression; Tac, Tacrolimus; CyA, Cyclosporine A; Th, Thalamus; Ln, Lentiform nucleus; pWM, parietal white matter; Cho, choline; Cr, creatine; Naa, N-acetyl-aspartate; mI, myo-Inositol; Glx, glutamine/glutamate; y, yes; n, no; IQR, interquartile range; na, not applicable; p value ≤0.05 is considered significant

10 of these 29 patients (51.4 ± 8.7 years, 100% male) studied after OLT had shown a PTE while 19 (52.7±10.0 years, 84% male) had not (Table 4). Additional surgical interventions were significantly more often performed in patients with PTE than in those without (p<0.001). Furthermore, all patients with a PTE were men (p<0.001). Otherwise the group characteristics showed no significant differences (Table 4). Both patient groups showed increased levels of Glx in the lentiform nucleus (p = 0.04). Patients without PTE after OLT showed significantly increased Cho levels in the lentiform nucleus (p = 0.02) compared to controls. Glx levels in the thalamus (p = 0.03) showed a significant difference between groups with higher levels in both patient groups than in controls, however, in post hoc analysis the level of significance was missed. No significant group differences were found concerning the white matter. The patient groups (PTE vs no PTE) did not significantly differ concerning brain metabolite levels about 15 months after OLT (Table 4).

**Table 4:** Characteristics and MRS values after OLT of patients with and without PTE.

	Patients with PTE n = 10 (Group 1)	Patients no PTE n = 19 (Group 2)	Controls n = 20 (Group 3)	p-value
**Age mean±SD**	51.4±8.7	52.7±10.0	47.8±10.8	0.30
**Gender male/female %**	10(100%)/0	16(84%)/3	6(30%)/14	*<0.001
**Underlying liver disease**	HCV n = 3 (30%) HBV n = 1 (10%) AI n = 1 (10%) AC n = 3 (30%) Other n = 2 (20%)	HCV n = 1 (5%) HBV n = 7 (36%) AI n = 3 (16%) AC n = 2 (11%) Other n = 6 (32%)	na	0.16
**Reason for OLT %**	chronic n = 7 (70%) ACLF n = 3 (30%)	chronic n = 17 (90%) ACLF n = 2 (10%)	na	0.31
**Additional surgery y/n %**	10(100%)	4(21%)/15	na	**<0.001**
**AH y/n %**	3(30%)/7	8(42%)/11	na	0.69
**DM y/n %**	2(20%)/8	3(16%)/16	na	1.00
**HC y/n %**	1(10%)/9	1(5%)/18	na	1.00
**GFR ml/min mean±SD**	81.0±30.8	75.3±37.7	-	0.69
**CKD ≥grade 3 y/n %**	3(30%)/7	7(37%)/12	na	1.00
**labMELD at OLT mean±SD**	19.4±7.8	19.3±10.6	na	0.67
**HE y/n %**	7(70%)/3	10(53%)/9	na	0.45
**WHC Grade %** **at OLT**	0 n = 6 (60%) 1 n = 3 (30%) 3 n = 1 (10%)	0 n = 14 (74%) 1 n = 4 (21%) 3 n = 1 (5%) 3 n = 1 (5%)	na	0.74
**IS Tac/CyA %** **after OLT**	7(70%)/3	9(47%)/10	na	0.43
**Th Cho mean±SD**	1.10±0.22	1.11±0.20	1.02±0.14	0.26
**Th Cr mean±SD**	3.32±0.38	3.36±0.40	3.41±0.39	0.82
**Th NAA mean±SD**	4.83±0.83	4.94±0.87	5.04±0.86	0.82
**Th mI mean±SD**	2.65±0.33	2.74±0.58	2.72±0.32	0.87
**Th Glx median (IQR)**	6.98 (2.10)	6.78 (1.18)	5.72 (1.86)	*0.03 **Group** 2 vs **Group** 3 0.06
**Ln Cho median (IQR)**	1.14 (0.43)	1.26 (0.42)	1.00 (0.12)	*<0.01 **Group 2 vs Group 3 <0.01**
**Ln Cr mean±SD**	3.86±0.75	4.06±0.86	3.83±0.36	0.55
**Ln NAA mean±SD**	4.90±0.66	5.08±0.89	4.96±0.47	0.78
**Ln mI median (IQR)**	2.50 (1.28)	2.83 (0.89)	2.72 (0.39)	0.38
**Ln Glx mean±SD**	8.01±1.47	7.88±2.00	6.57±0.86	*0.01 **Group 1 vs Group 3 0.04** **Group 2 vs Group 3 0.04**
**pWM Cho mean±SD**	0.98±0.23	1.09±0.26 (n = 18)	1.08±0.15 (n = 17)	0.42
**pWM Cr median (IQR)**	3.28 (0.56)	3.25 (0.46) (n = 18)	3.23 (0.26) (n = 17)	0.77
**pWM NAA median (IQR)**	5.37 (1.56)	5.39 (0.73) (n = 18)	5.60 (0.69) (n = 17)	0.57
**pWM mI mean±SD**	2.52±0.54	3.03±1.23 (n = 18)	3.08±0.63 (n = 17)	0.26
**pWM Glx median (IQR)**	5.07 (2.03)	4.64 (2.36) (n = 18)	4.91 (0.97) (n = 17)	0.88

MRS, magnetic resonance spectroscopy; OLT, liver transplantation; PTE, post-transplant encephalopathy; SD, standard deviation; n, number; HCV, Hepatitis C Virus; HBV, Hepatitis B Virus; AIH, Autoimmune Hepatitis; AC, alcoholic cirrhosis; ACLF, acute-on-chronic liver failure; AH, arterial hypertension, DM, diabetes mellitus, HC, hypercholesterolaemia; CKD, chronic kidney disease grade 3 according to GFR, glomerular filtration rate at the time of MRI/MRS examination; labMELD, laboratory Model of End Stage Liver Disease score; HE, hepatic encephalopathy; WHC, West Haven Criteria; IS, immunosuppression; Tac, Tacrolimus; CyA, Cyclosporine A; Th, Thalamus; Ln, Lentiform nucleus; pWM, parietal white matter; Cho, choline; Cr, creatine; Naa, N-acetyl-aspartate; mI, myo-Inositol; Glx, glutamine/glutamate; y, yes; n, no; IQR, interquartile range; na, not applicable; p value ≤0.05 is considered significant;

*, overall between groups

### Paired analysis before and after OLT

14 patients (age 49.2 ± 11.5 years, 85.7% male) completed the MRS measurements before (6.1±6.6 months) and after (12.1±3.6 months) OLT. The patient characteristics are displayed in Table 5. The mI levels significantly increased after OLT in the thalamus (p<0.01), lentiform nucleus (p = 0.04) and white matter (p = 0.01). Furthermore, Cho significantly increased after OLT in the thalamus (p = 0.04) and Glx significantly decreased in the white matter (p = 0.02) (Table 6).

**Table 5:** Characteristics of patients who underwent MRI before and after OLT.

	Patients n = 14	Patients with PTE n = 7	Patients no PTE n = 7	p-value
**Age mean±SD**	49.2±11.5	51.0±9.9	47.4±13.5	0.58
**Gender male/female %**	12(86%)/2	7(100%)/0	5(71%)/2	0.46
**Underlying liver disease**	HCV n = 3 (21%) HBV n = 2 (14%) AI n = 3 (21%) AC n = 2 (14%) Other n = 4 (30%)	HCV n = 2 (29%) HBV n = 0 AI n = 1 (13%) AC n = 2 (29%) Other n = 2 (29%)	HCV n = 1 (13%) HBV n = 2 (29%) AI n = 2 (29%) AC n = 0 Other n = 2 (29%)	0.32
**Reason for OLT %**	chronic 12 (86%) ACLF 2 (14%)	chronic 5 (71%) ACLF 2 (29%)	chronic 7 (100%)	0.46
**Additional surgery y/n %**	9(64%)/5	7(100%)	2(29%)/5	**0.02**
**AH y/n %**	2(14%)/12	1(14%)/6	1(14%)/6	1.00
**DM y/n %**	3(21%)/11	2(29%)/5	1(14%)/6	1.00
**HC y/n %**	0/14(100%)	-	-	-
**GFR ml/min mean±SD**	95.3±38.2	87.6±34.8	103±42.6	0.47
**CKD ≥grade 3 y/n %**	3(21%)/11	2(29%)/5	1(14%)/6	1.00
**labMELD at OLT** **mean±SD**	15.0±5.5	17.6±6.3	12.5±3.3	0.97
**History of HE y/n %**	5(36%)/9	4(57%)/3	1(14%)/6	0.26
**WHC Grade %** **at OLT**	0 n = 11 (79%) 1 n = 3 (21%)	0 n = 5 (71%) 1 n = 2 (29%)	0 n = 6 (86%) 1 n = 1 (14%)	1.00
**IS Tac/CyA %** **after OLT**	9(64%)/5	5(71%)/2	4(57%)/3	1.00

MRS, magnetic resonance spectroscopy; OLT, liver transplantation; PTE, post-transplant encephalopathy; SD, standard deviation; n, number; HCV, Hepatitis C Virus; HBV, Hepatitis B Virus; AIH, Autoimmune Hepatitis; AC, alcoholic cirrhosis; ACLF, acute-on-chronic liver failure; AH, arterial hypertension, DM, diabetes mellitus, HC, hypercholesterolaemia; CKD, chronic kidney disease grade 3 according to GFR, glomerular filtration rate at the time of MRI/MRS examination; labMELD, laboratory Model of End Stage Liver Disease score; HE, hepatic encephalopathy; WHC, West Haven Criteria; IS, immunosuppression; Tac, Tacrolimus; CyA, Cyclosporine A; y, yes; n, no; p value ≤0.05 is considered significant

**Table 6:** Paired MRS values of patients before and after OLT.

	Patients n = 14			Patients with PTE n = 7			Patients no PTE n = 7		
	before OLT	after OLT	p	before OLT	after OLT	p	before OLT	after OLT	p
**Th Cho** **median (IQR)**	**0.96 (0.17)**	**1.13 (0.37)**	**0.04**	0.92 (0.21)	0.99 (0.41)	0.31	0.97 (0.16)	1.20 (0.34)	0.09
**Th Cr** **median (IQR)**	3.59 (0.98)	3.46 (0.35)	0.43	3.60 (1.09)	3.37 (0.77)	0.50	3.57 (0.78)	3.59 (0.32)	0.61
**Th NAA** **median (IQR)**	4.36 (1.80)	5.30 (1.26)	0.36	4.02 (1.91)	5.01 (1.13)	0.74	4.96 (1.50)	5.44 (0.98)	0.50
**Th mI** **median (IQR)**	**1.46 (1.27)**	**2.83 (0.29)**	**<0.01**	**1.35 (0.74)**	**2.77 (0.73)**	**0.02**	**1.79 (1.79)**	**2.84 (0.27)**	**0.04**
**Th Glx** **median (IQR)**	8.02 (3.68)	6.78 (1.89)	0.13	10.3 (3.69)	6.65 (1.39)	0.13	7.89 (2.01)	6.90 (2.85)	0.61
**Ln Cho** **median (IQR)**	0.99 (0.35)	1.23 (0.40)	0.16	0.85 (0.35)	1.10 (0.35)	0.40	1.00 (0.24)	1.28 (0.19)	0.24
**Ln Cr** **median (IQR)**	4.24 (0.56)	4.06 (0.91)	0.27	4.22 (0.60)	3.56 (0.86)	0.24	4.26 (0.64)	4.17 (0.31)	0.61
**Ln NAA** **median (IQR)**	5.22 (1.33)	5.16 (0.87)	0.89	5.13 (1.26)	5.21 (0.76)	0.74	5.32 (1.96)	5.11 (1.21)	0.75
**Ln mI** **median (IQR)**	**1.98 (1.77)**	**2.79 (0.62)**	**0.04**	**1.17 (1.46)**	**2.47 (1.13)**	**0.04**	2.38 (2.01)	2.90 (0.67)	0.35
**Ln Glx** **median (IQR)**	9.48 (3.70)	8.29 (2.62)	0.06	**10.2 (4.54)**	**7.91 (3.00)**	**0.04**	8.35 (2.32)	8.75 (2.98)	0.61
**pWM Cho** **median (IQR)**	1.03 (0.26) n = 12	0.97 (0.38)	0.72	1.04 (0.28)	0.96 (0.42)	0.80	1.01 (0.28) n = 5	0.98 (0.45)	0.35
**pWM Cr** **median (IQR)**	3.30 (0.40) n = 12	3.19 (0.51)	0.88	3.37 (0.43)	3.26 (0.50)	0.74	3.23 (035) n = 5	3.09 (0.74)	0.69
**pWM NAA** **median (IQR)**	5.75 (0.73) n = 12	5.42 (1.08)	0.16	5.98 (0.63)	5.38 (1.49)	0.13	5.47 (0.43) n = 5	5.47 (0.77)	0.89
**pWM mI** **median (IQR)**	**1.30 (1.56)** n = 12	**2.63 (0.97)**	**<0.01**	**0.94 (1.27)**	**2.42 (0.99)**	**0.02**	1.47 (1.62) n = 5	3.03 (1.83)	0.23
**pWM Glx** **median (IQR)**	**5.89 (3.29)** n = 12	**4.87 (1.93)**	**0.02**	**7.33 (7.93)**	**4.75 (2.21)**	**0.03**	5.68 (1.31) n = 5	5.58 (1.78)	0.69

MRS, magnetic resonance spectroscopy; OLT, liver transplantation; PTE, post-transplant encephalopathy; n, number; Th, Thalamus; Ln, Lentiform nucleus; pWM, parietal white matter; Cho, choline; Cr, creatine; Naa, N-acetyl-aspartate; mI, myo-Inositol; Glx, glutamine/glutamate; IQR, interquartile range; p value ≤0.05 is considered significant

**Fig 4:**
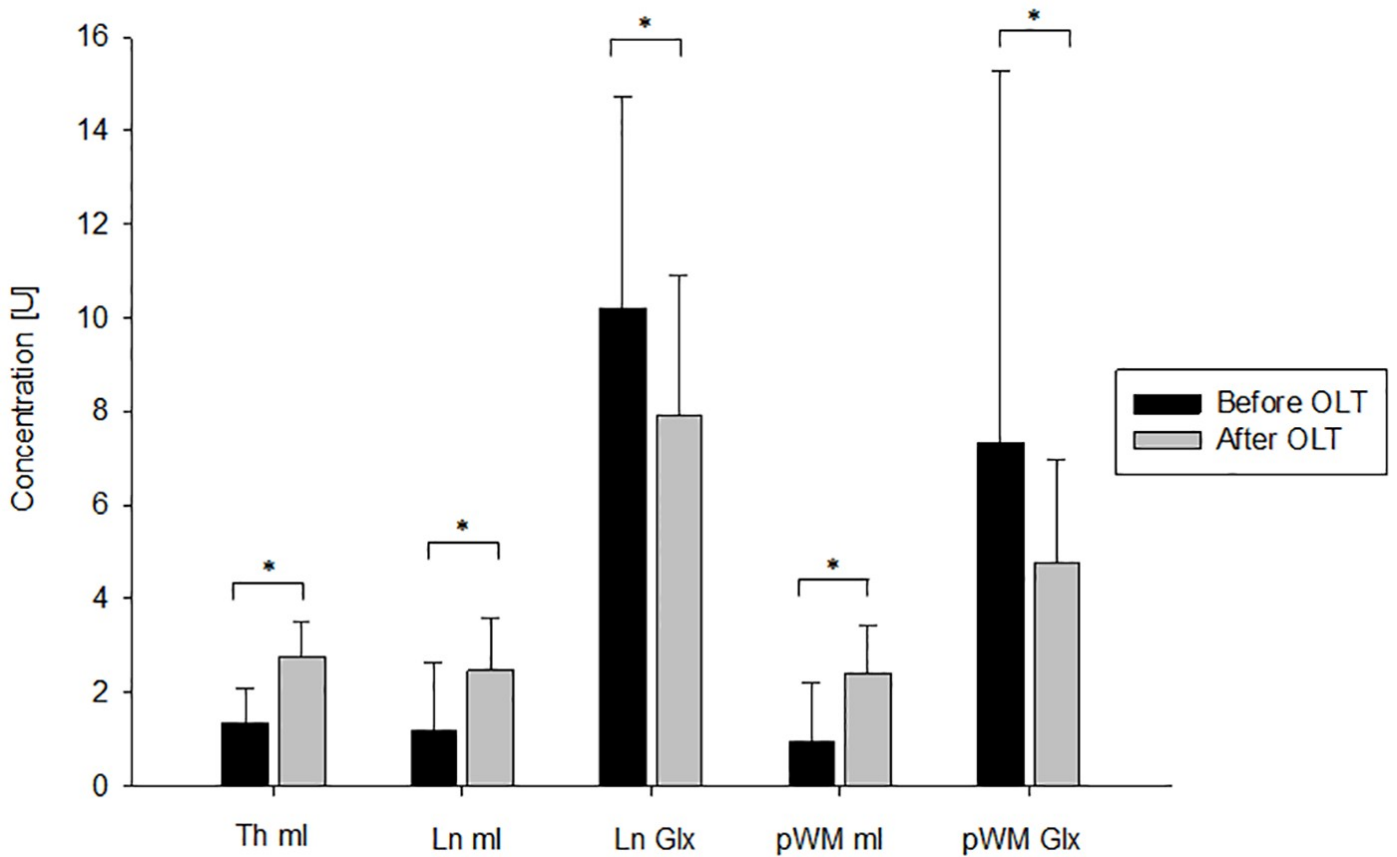
Change of MRS values from before to after OLT in patients with PTE. This figure illustrates the significant changes of cerebral metabolite concentrations from before to after OLT in the 7 patients that developed a PTE. * p<0.05; Th, Thalamus; Ln, Lentiform nucleus; pWM, parietal white matter; mI, myo-Inositol; Glx, glutamine/glutamate; OLT, liver transplantation; U, arbitrary unit.

**Fig 5:**
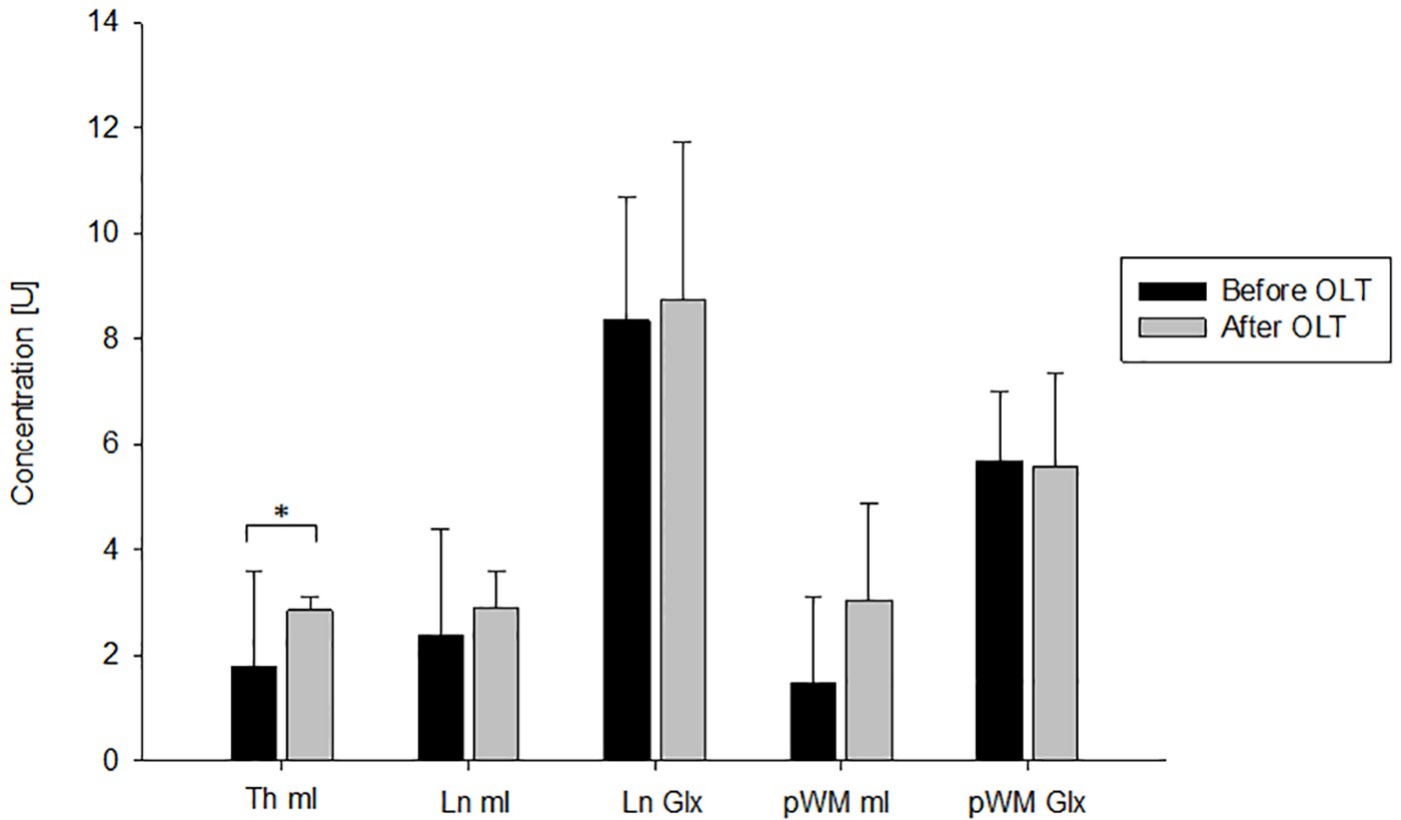
Change of MRS values from before to after OLT in patients without PTE. This figure illustrates the changes of cerebral metabolite concentrations from before to after OLT in the 7 patients without PTE. * p<0.05; Th, Thalamus; Ln, Lentiform nucleus; pWM, parietal white matter; mI, myo-Inositol; Glx, glutamine/glutamate; OLT, liver transplantation; U, arbitrary unit.

Seven (50%) patients (age 51.0±9.9 years, 100% male) developed a PTE. They underwent MRI 7.9±8.5 months before and 10.9±4.7 months after OLT. Again additional surgical interventions after OLT were more frequent in patients with a PTE after OLT (p = 0.02) (Table 5). Interestingly, the patients with PTE showed more pronounced alterations of the brain metabolite levels before OLT compared to those without PTE (Table 6 and Figs 4 and 5). The mI levels significantly increased in the thalamus (p = 0.02), lentiform nucleus (p = 0.04) and white matter (p = 0.02) after OLT and the Glx levels significantly decreased in the lentiform nucleus (p = 0.04) and white matter (p = 0.03) (Table 6 and Fig 4). The 7 patients without PTE (age 47.4±13.5 years, MRI 4.3±4.0 months before and 13.4±1.5 months after OLT) only showed a significant change of the mI level in the thalamus (p = 0.04, Fig 5). Additionally, for each metabolite the ratio to creatine was calculated. The results of the paired analysis are displayed in Table 7. The results of the ratios support the results of the absolute values. Interestingly, more regions showed significant changes. However, the patients with PTE after OLT still showed more pronounced restitution of brain metabolites than patients without PTE.

**Table 7:** Paired analysis of metabolite /creatine ratios before and after OLT.

	Patients n = 14			Patients with PTE n = 7			Patients no PTE n = 7		
	before OLT	after OLT	p	before OLT	after OLT	p	before OLT	after OLT	p
**Th Cho** **median (IQR)**	**0.26 (0.05)**	**0.33 (0.04)**	**<0.01**	0.25 (0.11)	0.32 (0.05)	0.08	**0.26 (0.04)**	**0.34 (0.06)**	**0.03**
**Th NAA** **median (IQR)**	**1.29 (0.26**	**1.50 (0.17)**	**0.03**	1.29 (0.35)	1.50 (0.20)	0.31	**1.28 (0.16)**	**1.61 (0.15)**	**0.03**
**Th mI** **median (IQR)**	**0.36 (0.39)**	**0.81 (0.09)**	**0.001**	**0.34 (0.37)**	**0.81 (0.11)**	**0.02**	**0.51 (0.50)**	**0.81 (0.02)**	**0.02**
**Th Glx** **median (IQR)**	2.20 (0.81)	1.94 (0.60)	0.12	**2.61 (0.74)**	**1.96 (0.73)**	**0.04**	1.91 (0.38)	1.91 (0.62)	0.92
**Ln Cho** **median (IQR)**	**0.26 (0.08)**	**0.31 (0.04)**	**0.002**	**0.22 (0.07)**	**0.30 (0.04)**	**0.03**	**0.25 (0.06)**	**0.31 (0.03)**	**0.03**
**Ln NAA** **median (IQR)**	1.16 (0.28)	1.33 (030)	0.05	**1.12 (0.20)**	**1.36 (0.15)**	**0.04**	1.23 (0.40)	1.21 (0.20)	0.35
**Ln mI** **median (IQR)**	**0.48 (0.31)**	**0.68 (0.10)**	**<0.01**	**0.30 (0.35)**	**0.65 (0.09)**	**0.03**	0.53 (0.37)	0.70 (0.18)	0.91
**Ln Glx** **median (IQR)**	2.20 (0.62)	2.05 (0.58)	0.14	2.54 (0.74)	2.30 (0.47)	0.09	1.95 (0.57)	1.92 (0.67)	0.80
**pWM Cho** **median (IQR)**	0.32 (0.09) n = 12	0.32 (0.07)	0.88	0.31 (0.10)	0.28 (0.09)	0.87	0.32 (0.10) n = 5	0.32 (0.07)	0.68
**pWM NAA** **median (IQR)**	1.84 (0.35) n = 12	1.69 (0.37)	0.12	1.84 (0.37)	1.67 (0.30)	0.13	1.70 (0.33) n = 5	1.70 (0.44)	0.50
**pWM mI** **median (IQR)**	**0.39 (0.52)** **n = 12**	**0.90 (0.31)**	**0.01**	**0.19 (0.44)**	**0.76 (0.35)**	**0.02**	0.45 (0.58) n = 5	0.92 (0.43)	0.23
**pWM Glx** **median (IQR)**	**1.84 (1.28)** **n = 12**	**1.54 (0.45)**	**0.02**	**2.61 (1.32)**	**1.53 (0.61)**	**0.02**	1.81 (0.47) n = 5	1.54 (0.40)	0.69

MRS, magnetic resonance spectroscopy; OLT, liver transplantation; PTE, post-transplant encephalopathy; n, number; Th, Thalamus; Ln, Lentiform nucleus; pWM, parietal white matter; Cho, choline; Cr, creatine; Naa, N-acetyl-aspartate; mI, myo-Inositol; Glx, glutamine/glutamate; IQR, interquartile range; p value ≤0.05 is considered significant

## Discussion

This prospective single center study evaluated whether cerebral metabolite changes in patients with liver cirrhosis before OLT are associated with the occurrence of a PTE in the first weeks after OLT. Indeed, before OLT both patient groups, those with and those without PTE showed the well-known alterations of brain metabolite levels characteristic for patients with liver cirrhosis: an increase of Glx and a decrease of Cho and mI concentrations [10, 18, 19]. These alterations, however, were more pronounced in the patients who developed PTE than in those who did not.

The characteristic alterations of brain metabolite levels in patients with liver cirrhosis are believed to be caused by an increased ammonia uptake into the brain. Astrocytes process ammonia and metabolize glutamate to glutamine. The increase of glutamine activates an osmotic compensatory mechanism through which water enters the cell. The resulting cell swelling is further increased by oxidative stress and inflammation which impair the cell membrane integrity. In consequence to the osmotic pressure and damaged cell membrane myo-Inositol besides other osmolytes leaves the cell [10, 11].

These brain metabolite alterations were even described to correlate with the grade of HE [18, 20]. However, alterations of brain metabolites in patients with liver cirrhosis were also seen in patients without HE [9, 21, 22]. Furthermore, cerebral metabolite changes recover within months after liver transplantation, both in patients with and without a history of HE [9].

In the first weeks after OLT up to 30% of the patients develop disorientation, confusion, clouding of consciousness, hallucinations or seizures [3, 23]. A PTE is diagnosed after the exclusion of structural brain lesions and dysfunction of the transplanted liver. PTE is a metabolic-toxic induced neurological complication which in contrast to HE is not caused by liver dysfunction. The exact pathomechanism of PTE is unknown so far, however, several factors are discussed: cerebral metabolite changes before OLT connected to chronic liver dysfunction [5, 12, 24], shift of cerebral electrolytes during and after surgery [5] as well as an effect of calcineurin inhibitors used for immunosuppression after OLT [2].

Dhar and colleagues showed that patients with a history of HE—and especially those suffering from HE directly before OLT—had an increased risk to develop encephalopathy after OLT [24]. Furthermore, prolonged and repeated surgeries as well as ACLF were identified as independent risk factors for the development of metabolic-toxic induced neurological complications after OLT [3]. Prolonged and repeated metabolic alterations during and after surgery might increase the vulnerability of the CNS towards metabolic-toxic influences in case of pre-existing cerebral damage [3]. Also, after OLT the immunosuppressive therapy usually consists of calcineurin inhibitors which were shown to be neurotoxic. In human cells as well as in rat brain models CNI inhibit the mitochondrial energy metabolism, induce oxidative stress and interfere with the NO-metabolism [6, 7, 25–27]. Furthermore, CNI therapy was associated with the development of neurological complications in the first weeks after OLT [2] and long-term cognitive dysfunction [28].

These observations led to our hypothesis that alterations of cerebral metabolites are involved in the development of PTE after OLT.

In the subset of patients who underwent MRS before and after OLT we were able to show that especially patients who developed PTE had significant alterations of Glx and mI concentrations before OLT which normalized considerably after OLT. In line with this observation in this small sub-group also the MRS data of the patients with PTE who could be examined before OLT showed more pronounced alterations of the brain metabolite levels than those without PTE though the level of significance was missed probably due to the small patient numbers. In patients without PTE the brain metabolite alterations before OLT were less severe compared to patients with PTE. Thus, significantly altered cerebral metabolites in patients with liver cirrhosis might be a risk factor for the development of PTE after OLT. The lack of significant differences comparing patients with and without PTE might be due to low patient numbers, predominantly, but it could also hint to a tolerance threshold for metabolite alterations that may not be exceeded without clinical impact. In addition, it has to be considered that MRS was performed several months before and after OLT, respectively. Thus, the concentrations of cerebral metabolites analyzed in this study may be different from those at the time of OLT and the first weeks thereafter. Still, the values measured before OLT can be considered representing the extent of metabolic changes in the individual patient. This assumption is backed up by a former study by Ahluwalia and colleagues who showed a remarkable consistency of brain MRS alterations in patients with liver cirrhosis in a follow-up study repeating the MRS about 11 months after the first examination [29]. For the time after OLT changes of cerebral metabolite concentrations have to be expected. Differences between patients with and without PTE might be present within the first days and weeks after transplantation, and align thereafter. This should be clarified in future studies.

Of course, other factors besides preexisting cerebral metabolite alterations may be involved in the development of PTE. A history of HE was classified as a risk factor for the development of encephalopathy in the first weeks after OLT [24, 30]. Probably due to the small number of patients considered we found no significant group difference between patients with and without PTE in our study cohort concerning HE history, especially in the subgroup of patients that underwent MRS before and after OLT. However, of 7 patients who developed PTE after OLT 4 (57%) had a history of HE in contrast to only 1 (14%) of 6 in the group of patients that did not develop PTE after OLT. This observation is not surprising considering the fact that the development of HE is also related to osmolyte extrusion from the astrocytes. Yet, some patients without a history of HE develop a PTE after OLT and not all patients with a history of HE seem to develop a PTE after OLT (Tables 2, 4 and 5). The number of additional surgical interventions after OLT was significantly higher in patients who developed a PTE. This was described previously [3]. We found no significant difference between our patient groups concerning ACLF induced OLT. This might, however, as well be a consequence of the patient numbers. All patients included into this study received CNI therapy for immunosuppression. Currently this is the standard immunosuppressive therapy regimen after OLT [31] and thus it is impossible to analyze the effect of CNI upon the development of PTE after OLT.

Overall, a PTE after OLT is unlikely to be caused by only one factor. Rather the combination of deranged cerebral metabolites connected to liver dysfunction or ACLF, repeated surgical interventions after OLT as well as CNI induced neurotoxicity increase the risk to develop a PTE after OLT.

Interestingly, Cr concentrations were higher in the thalamus (p = 0.04) and lentiform nucleus (p<0.001) in patients who did not develop PTE after OLT compared to controls (Table 2). Cr concentrations were shown to change in disease and are elevated in case of neuroinflammation and glial activation [32, 33]. Cr is part of the energy metabolism via the creatine kinase [34] and was shown to be neuroprotective in animal models [35, 36]. Thus, our results indicate that patients who do not develop PTE might have a glial activation in the grey matter and support the assumption that elevated Cr concentrations might be neuroprotective. This might be explained by an increased energy buffer which compensates for possible energy metabolism impairment after transplantation. Consequently, our results indicate that higher Cr concentrations in the grey matter might protect against the development of PTE after OLT.

Our healthy controls showed a positive correlation of brain mI concentrations with age which was previously described [37]. Interestingly, patients showed a decline of brain mI levels with age. This might indicate that older patients are more susceptible towards liver dysfunction induced brain metabolite alterations considering that brain metabolites change with age [38] and that age is a risk factor for the development of HE in cirrhotic patients [39].

There is one other observation in our study that has not been described before: the significantly increased levels of Glx and choline in the deep grey matter after OLT compared to healthy controls. It is impossible to differentiate between glutamate and glutamine in a 1.5 T MRS study, thus interpretation of this finding is compromised. Furthermore, elevated Cho levels after OLT indicate increased cellular membrane turnover. Both observations are worth to be further elucidated.

The limitations of our single center study comprise the low patient numbers and the difference in sex ratios between patient and control groups. Despite the latter limitation our patient cohort showed similar alterations of cerebral metabolites as described in the literature. Thus, a significant sex effect is not expected and our study cohort can be assumed to be representative. Furthermore, MRS measurements were performed several months before and after OLT. Thus, brain metabolite concentrations discussed here might be significantly different from those directly before and in the first weeks after OLT. Former studies, however, showed a remarkable consistency of MRS alterations in patients with liver cirrhosis in a follow-up study about 11 months apart from the baseline study [29] underscoring the suitability of our data for the conducted analyses. Concerning the methodology, partial volume correction was not available. Thus, the pWM VOI might include small amounts of cerebrospinal fluid and grey matter. Furthermore, FWHM line widths above 0.1 ppm might reduce the quality of the shimming for the spectroscopy sequences.

In conclusion, comparison of brain metabolites before OLT showed distinct though not significant differences between patients with and without PTE. Analysis of the small sub-group of patients who underwent MRS before and after OLT, however, showed significant differences between those with and without PTE and thus indicated that altered cerebral metabolites in patients with liver cirrhosis might be a risk factor for the development of PTE after OLT.

## Supporting information

S1 Dataset This Dataset contains all data underlying our results. (XLSX)

## Abbreviations

^1^H-MRS: proton magnetic resonance spectroscopy
ACLF: acute-on-chronic liver failure
AH: arterial hypertension
AIH: autoimmune hepatitis
ALF: acute liver failure
Cho: choline
CI: confidence interval
CKD: chronic kidney disease grade III
CLD: chronic liver disease
CNI: calcineurin inhibitor
CNS: central nervous system
Cr: creatine/phosphocreatine
CyA: cyclosporine A
DM: diabetes mellitus
FWHM: Full Width at Half Maximum
GFR: glomerular filtration rate ml/min
Glx: glutamine/glutamate
HBV: Hepatitis B Virus
HC: hypercholesterolemia
HCV: Hepatitis C Virus
HE: hepatic encephalopathy
IQR: interquartile range
IS: immunosuppression
labMELD: laboratory Model of End Stage Liver Disease score
Ln: Lentiform nucleus
mI: myo-Inositol
MRI: magnetic resonance imaging
MRS: magnetic resonance spectroscopy
NAA: N-acetyl-aspartate/N-acetyl-aspartate-glutamate
OLT: orthotopic liver transplantation
PBC: primary biliary cirrhosis
PLD: polycystic liver disease
PSC: primary sclerosing cholangitis
pWM: parietal white matter
Tac: tacrolimus
Th: thalamus
WHC: West Haven Criteria
